# A Simplified Method for Three-Dimensional (3-D) Ovarian Tissue Culture Yielding Oocytes Competent to Produce Full-Term Offspring in Mice

**DOI:** 10.1371/journal.pone.0143114

**Published:** 2015-11-16

**Authors:** Carolyn M. Higuchi, Yuuki Maeda, Toshitaka Horiuchi, Yukiko Yamazaki

**Affiliations:** 1 Institute of Biogenesis Research, John A. Burns School of Medicine, University of Hawaii, Honolulu, Hawaii, United States of America; 2 Graduate School of Comprehensive Scientific Research, Prefectural University of Hiroshima, Hiroshima, Japan; Qingdao Agricultural University, CHINA

## Abstract

*In vitro* growth of follicles is a promising technology to generate large quantities of competent oocytes from immature follicles and could expand the potential of assisted reproductive technologies (ART). Isolated follicle culture is currently the primary method used to develop and mature follicles *in vitro*. However, this procedure typically requires complicated, time-consuming procedures, as well as destruction of the normal ovarian microenvironment. Here we describe a simplified 3-D ovarian culture system that can be used to mature multilayered secondary follicles into antral follicles, generating developmentally competent oocytes *in vitro*. Ovaries recovered from mice at 14 days of age were cut into 8 pieces and placed onto a thick Matrigel drop (3-D culture) for 10 days of culture. As a control, ovarian pieces were cultured on a membrane filter without any Matrigel drop (Membrane culture). We also evaluated the effect of activin A treatment on follicle growth within the ovarian pieces with or without Matrigel support. Thus we tested four different culture conditions: C (Membrane/activin-), A (Membrane/activin+), M (Matrigel/activin-), and M+A (Matrigel/activin+). We found that the cultured follicles and oocytes steadily increased in size regardless of the culture condition used. However, antral cavity formation occurred only in the follicles grown in the 3-D culture system (M, M+A). Following ovarian tissue culture, full-grown GV oocytes were isolated from the larger follicles to evaluate their developmental competence by subjecting them to *in vitro* maturation (IVM) and *in vitro* fertilization (IVF). Maturation and fertilization rates were higher using oocytes grown in 3-D culture (M, M+A) than with those grown in membrane culture (C, A). In particular, activin A treatment further improved 3-D culture (M+A) success. Following IVF, two-cell embryos were transferred to recipients to generate full-term offspring. In summary, this simple and easy 3-D ovarian culture system using a Matrigel drop and activin A supplementation (M+A) provides optimal and convenient conditions to support growth of developmentally competent oocytes *in vitro*.

## Introduction

In the mammalian ovary, folliculogenesis is critically regulated to provide fertilizable oocytes for the duration of a female’s reproductive life. In vitro growth (IVG) of mammalian follicles could be a promising tool for use with assisted reproductive technologies (ART) to derive large quantities of fertilizable oocytes derived from immature follicles. Moreover, this system could provide a valuable research tool for further elucidation of the poorly understood molecular mechanisms of folliculogenesis. Therefore, establishing an in vitro follicle culture model that can accurately mimic the in vivo ovarian growth environment has been crucial. Until now many different approaches have been used with varying success, however, a single standard protocol has yet to be established, even in a model species such as the mouse [1]. The difficulties of in vitro follicle development originate in the unique features of the follicle. Individual follicles within the ovary feature a vascular system built up around a 3-D structure in which the oocyte is surrounded by follicular cells [1]. Growth of the oocyte is critically dependent on autocrine and paracrine bidirectional signaling between the oocyte and the surrounding follicular cells [2–3]. Due to the requirement for this crosstalk between the oocyte and follicular cells, maintenance of follicle architecture during the growth process is crucial for the correct acquisition of developmental competence of oocytes.

In general, ovarian tissue culture as a technology for in vitro follicle development has not been highly effective. While primordial follicle growth can be supported in this system, proper development of follicles beyond the pre-antral stage has been inhibited [4]. Instead of culturing the tissue itself, individual follicles have been isolated from the ovary and cultured on a dish/membrane to maintain their proper development (2-D follicle culture). Actually, the majority of early groundbreaking work on in vitro follicle culture was undertaken using conventional 2-D follicle culture systems. Eppig and Schroeder (1989) were able to obtain live offspring after in vitro culture of isolated mouse pre-antral follicles on collagen gel and the use of IVF to fertilize these IVM oocytes [5]. This same group further reported that primordial follicles can be cultured to complete maturation using a combination of ovarian tissue culture and 2-D follicle culture to generate developmentally competent oocytes [6]. In general, 2-D follicle culture systems have utilized a 2-D surface (dish /membrane) coated with extracellular matrix (ECM) proteins [7–10]. In mammalian ovaries, ECM proteins play key roles in structural support, cell aggregation and communication, cell survival, proliferation, and biochemical signal regulation [9, 11–12]. For example, laminin and type IV collagen have been observed in the basal lamina of follicles in several different species [13–16], suggesting addition of ECM proteins to 2-D follicle cultures may help mimic normal environmental signaling that occurs in vivo to promote follicle growth in 2-D culture systems. However, it has been recognized that 2-D follicle culture is not sufficient to sustain the normal architecture of follicles in vitro [1, 4, 17].

In recent years, further technical advancement has emerged in the form of 3-D follicle culture, which better sustains normal follicle structure in vitro. In 3-D follicle culture systems, isolated follicles are individually encapsulated in various types of substrates such as collagen, alginate, hyaluronan hydrogel, PEG, or Matrigel to maintain the normal 3-D architecture of the follicle in vitro [1, 4, 17]. To date, encapsulation with alginate gel has been the most widely applied system for 3-D follicle culture [4, 17]. For example, secondary—preantral follicles encapsulated with alginate gel showed growth in vitro [18–20], and resulted in the production of live offspring from oocytes grown under these 3-D follicle culture conditions [21]. However, 3-D follicle culture is typically considered a complex and time-consuming procedure. For example, the protocol for the culture of murine follicles in an alginate matrix typically involves the following steps–dissociation of the ovary to isolate follicles, selection of healthy follicles, placement of single follicles into each alginate droplet, immersion of alginate + follicle complexes in calcium chloride solution to crosslink the alginate, and culture of encapsulated follicles in individual wells of a 96-well plate [19–23].

Here we describe a simple and easy 3-D ovarian tissue culture system for growth and maturation of follicles from mice. To create a more robust *in vivo*-like ovarian culture system, we utilized a solid Matrigel drop (1:1 dilution with culture medium) to support the proper 3-D structure of follicles in the ovarian tissue. An ovary was cut into 8 pieces and one piece of the tissue was placed on top of each Matrigel drop supported on a membrane. This procedure is much simpler than either the 2-D or 3-D isolated follicle culture systems described above. Because our goal was to generate fertilizable follicular oocytes in vitro, the developmental competence of the resulting oocytes was compared to that of oocytes derived from follicles cultured on membranes without Matrigel drop. We also evaluated the effect of activin A treatment on follicle growth in ovarian tissue cultured with or without Matrigel support. Activin A, a member of the transforming growth factor β (TGF-β) superfamily, is normally generated by granulosa cells in primary to antral stage follicles [24] and regulates proliferation, differentiation, and steroidogenesis within the follicles [25]. Despite evidence from previous studies indicating that activin A treatment enhances preantral follicle development and oocyte growth in various species such as mouse [26–29], human [30] and sheep [31], it has not been clear how activin A treatment affects the quality of oocytes grown in vitro. Here we describe a new technology for IVG of follicles that yields high quality oocytes competent to yield live offspring after IVF, thus validating the utility of the oocytes produced by this simple and easy ovarian tissue culture method.

## Materials and Methods

### Mice

B6D2F1 (C57BL/6 x DBA/2) female mice were mated with B6D2F1 males to obtain (B6D2F1 x B6D2F1) F2 female pups. Prepubertal ovaries were collected from these F2 females at 14 days post partum (14 dpp). For IVF, sperm were obtained by dissection of the cauda epididymides of adult B6D2 F1 male mice. CD-1 female mice were used as surrogate mothers after mating with vasectomized CD-1 males. All relevant experimental protocols were reviewed and approved by the Institutional Animal Care and Use Committee of the University of Hawaii.

### Media

Ovarian tissue culture was performed using in vitro growth (IVG) medium consisting of αMEM (Invitrogen, CA) supplemented with 5% FBS, 100 mIU/ml rhFSH (MERCK, NJ), 5 μg/ml insulin, 5 μg/ml transferrin, 5 ng/ml selenium (Sigma-Aldrich, MO). Activin A (Sigma-Aldrich, MO) was used as an IVG supplement (30 ng/ml). IVM medium consisted of αMEM supplemented with 5% FBS, 100 mIU/ml rhFSH and 5 ng/ml rhEGF (Promega, WI). TYH medium was used for IVF. After IVF, fertilized oocytes and embryos were cultured in mCZB medium.

### Ovarian tissue culture

Ovaries at 14 dpp were cut into 8 pieces and subjected to organ culture as described below. As a control, ovarian tissue pieces were directly placed onto a floating membrane filter (0.4 μm HTTP; Millipore, MA) (4 pieces/membrane) on IVG medium and cultured with or without activin A treatment (Membrane culture). Matrigel is a soluble extract of basement membrane protein derived from the Engelbreth-Holm-Swarm tumor that forms a 3-D gel at 37°C and supports cell morphogenesis, differentiation, and tumor growth [32]. For our 3-D culture system, Matrigel (BD Biosciences, MA) was diluted with IVG medium (1:1) and 5 μl were placed on a floating membrane filter to make a small Matrigel drop (4 drops/membrane). Then, a single ovarian tissue piece was placed on top of each drop (1 piece/drop) and cultured with or without activin A treatment. Each ovarian tissue piece partially sank into the Martigel but the top surface of each piece retained access to surrounding air. We tested four different culture conditions: Control (C: Membrane/activin-), with activin A (A: Membrane/activin+), with Matrigel but no activin A (M: Matrigel/activin-), and with Matrigel and activin A (M+A: Matrigel/activin+). Ovarian tissues were cultured under each the four conditions for 10 days at 37°C, 5%CO_2_ in air. Half of the culture media was replaced every other day.

### Follicle and oocyte growth

On Days 0, 6, and 10 of ovarian tissue culture, growing follicle diameter and oocyte diameter were measured. Under a stereomicroscope, the tissues were dissected to isolate well-advanced individual follicles. Follicles were visualized using an inverted microscope (Olympus, PA) and measured with an ocular micrometer calibrated at 400x magnification. The longest and shortest diameters of each follicle were averaged to yield the respective value. These follicles were then punctured to obtain germinal vesicle (GV) stage oocytes surrounded by granulosa cells. To measure oocyte size, surrounding granulosa cells were mechanically removed by gentle pipetting. Each oocyte diameter was measured as an average of the longest and shortest dimensions at 400X magnification.

### Classification of oocyte GV chromatin configurations

Chromatin in the GV oocyte is initially decondensed with the nucleolus not surrounded by heterochromatin (Non Surrounded Nucleolus: NSN). During oocyte growth, the GV chromatin condenses into perinucleolar rings (Surrounded Nucleolus: SN) [33]. To determine the status of the chromatin in GVs, we isolated oocytes from well-advanced follicles on Days 0, 6, and 10 of ovarian tissue culture. After being freed from the surrounding granulosa cells, the oocytes were treated with Hoechst dye (1:1000) for 8 minutes at 37°C. Under a fluorescence microscope, the oocytes were categorized into three chromatin configurations: NSN, SN, and an intermediate stage between NSN and SN configurations (Int).

### Histological analysis

On Days 0, 6, and 10 of ovarian tissue culture, tissues were fixed overnight in Bouin’s solution and subsequently treated with ascending concentrations of ethanol (70–100%) and xylene. Serial 5 μm paraffin sections were cut and stained with hematoxylin and eosin.

### *In vitro* maturation (IVM)

After 10 days of ovarian tissue culture, full-grown GV oocytes were subjected to IVM. Largely grown follicles were punctured to obtain GV oocytes surrounded by granulosa cell layers. These oocytes with granulosa cells or cumulus cells were cultured in IVM media for 15–17 hrs at 37°C, 5% CO_2_ in air. After culture, oocytes with first polar bodies were recognized as matured metaphase II (MII) oocytes.

### *In vitro* fertilization (IVF)

After IVM, in vitro matured oocytes with expanded granulosa cells/cumulus cells were subjected to IVF. Sperm mass collected from the caudal epididymides of adult males were incubated in TYH medium at 37°C, 5% CO_2_ in air. After 1 hr of pre-incubation, a small aliquot of sperm suspension was added to a TYH drop containing oocytes for IVF. Six hrs after IVF, oocytes were washed and transferred into mCZB media. Twenty-four hrs after IVF, embryo development to the 2-cell stage was assessed.

### Embryo transfer (ET)

Embryos at the 2-cell stage were transferred into the oviducts of surrogate mothers that had been mated with vasectomized males the previous night (= Day 0.5 of gestation). On Day 19.5 of gestation, full-term pups were delivered by Cesarean section or naturally delivered. In vivo control pups were derived from natural mating between B6D2F1 female and male mice. Body weight and placenta weight of each pup were measured.

### Statistical analysis

Follicle and oocyte sizes were analyzed by variance analysis (one-way multiple comparison ANOVA) and the differences were subjected to Tukey’s test. Chi-square testing was used to analyze differences in oocyte chromatin configuration, IVM, IVF, and ET rates.

## Results

### Follicle and oocyte growth

**Fig 1:**
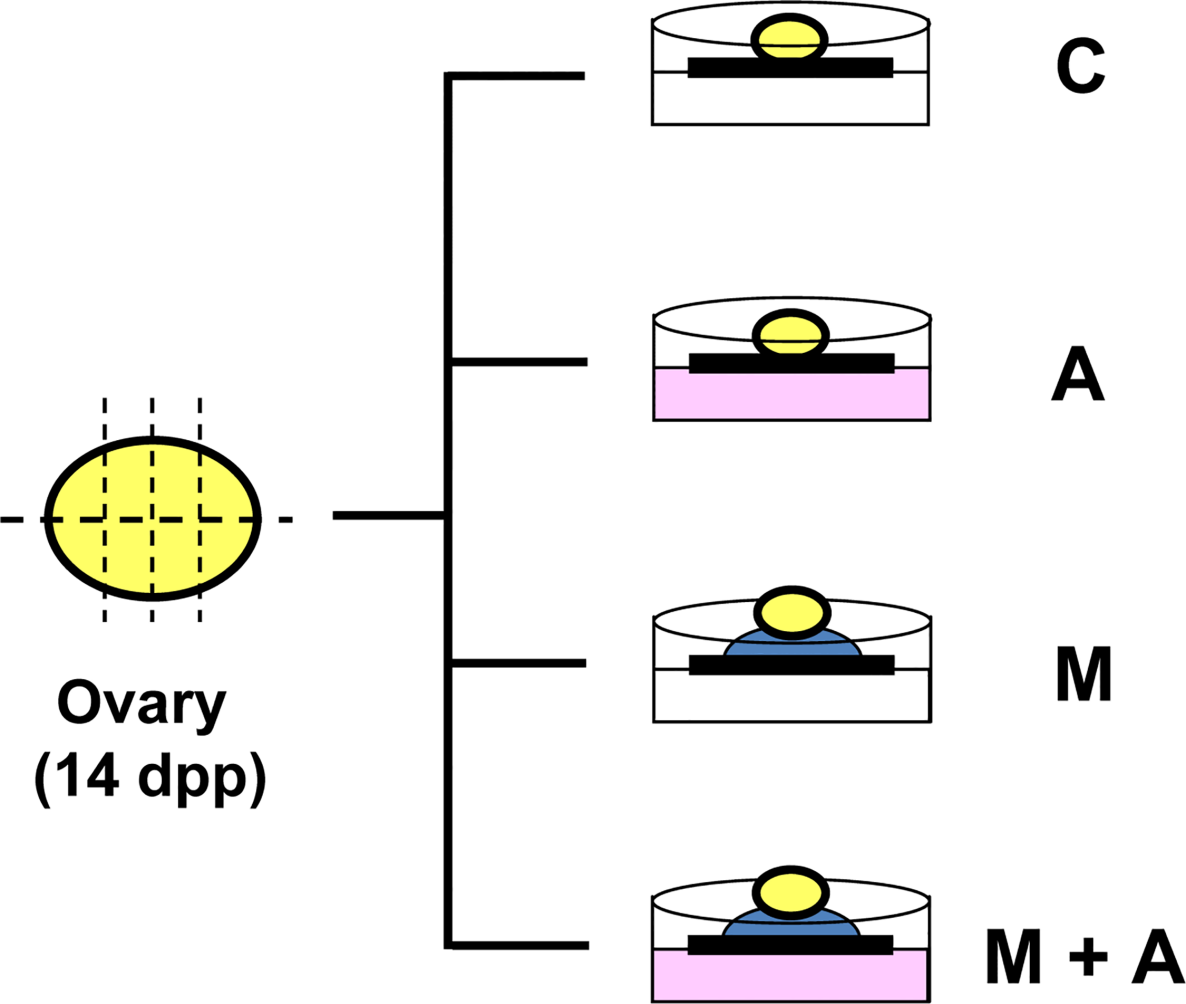
Ovarian tissue culture under 4 different culture conditions. A mouse ovary at 14 dpp was cut into 8 pieces and subjected to organ culture. Ovarian tissue pieces were directly placed onto a floating membrane filter on IVG medium and cultured with or without activin A (Membrane culture). For our 3-D culture system, Matrigel diluted with IVG medium (1:1) was placed on a membrane filter to make a Matrigel drop. A single ovarian tissue was placed on top of each drop and cultured with or without activin A treatment. The tissues were cultured for 10 days under four different culture conditions: Control (C: Membrane/activin-), with activin A (A: Membrane/activin+), with Matrigel but no activin A (M: Matrigel/activin-), and with Matrigel and activin A (M+A: Matrigel/activin+).

**Fig 2:**
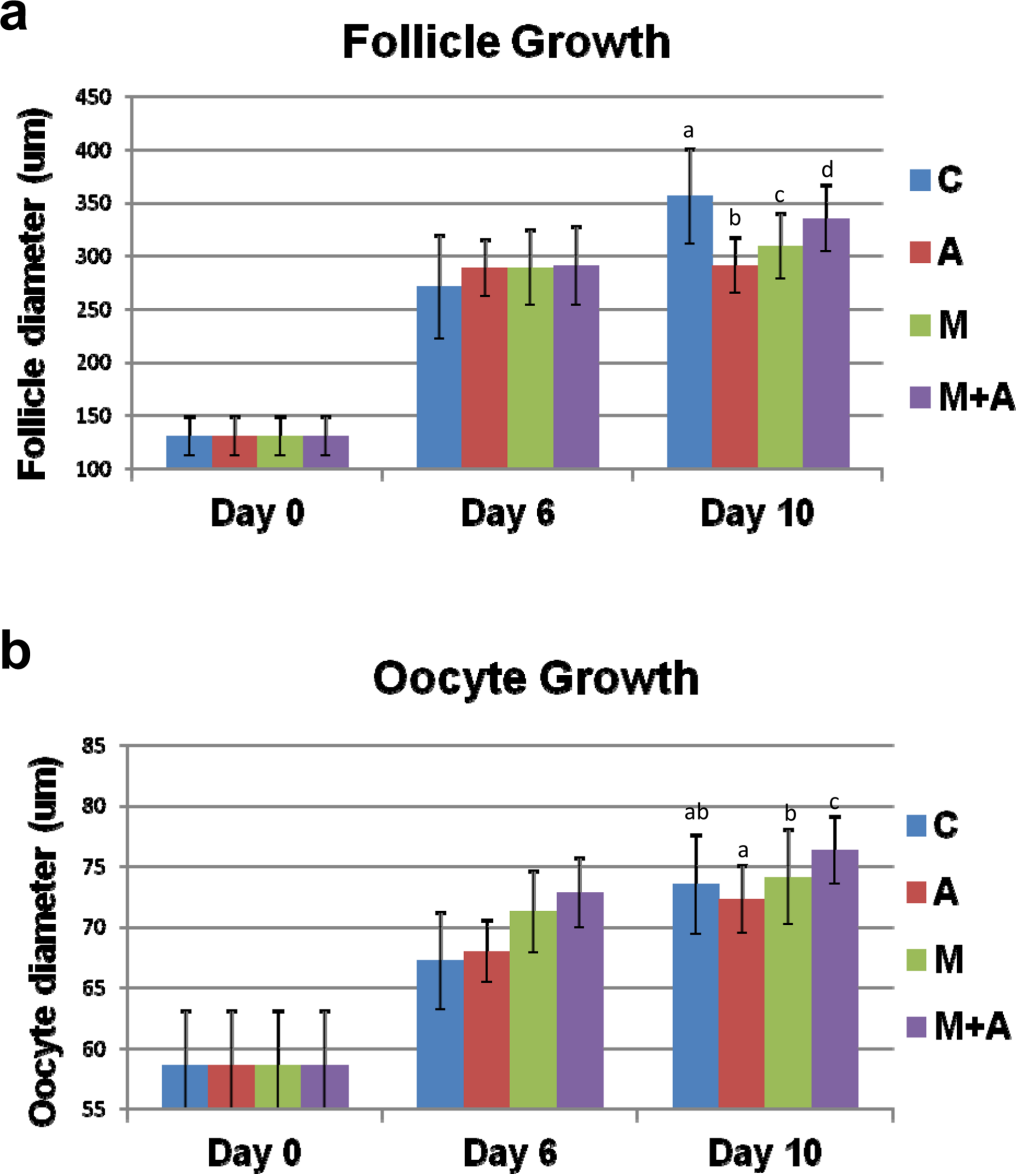
Follicle and oocyte growth in ovarian tissues cultured under 4 different conditions. **(a, b)** The ovarian tissues isolated from 14 dpp mice were cultured under 4 different conditions (C, A, M, M+A). On Days 0, 6 and 10 of culture, the growing follicle diameters (a) were measured. In all culture conditions, the advanced follicles increased their sizes on Day 6 (C vs A, M, M+A; *P*<0.01–0.05). On Day 10, the follicles reached 299–356 μm in all conditions (C vs A, M, M+A, A vs M, M+A, M vs M+A; *P*<0.05)., On Days 0, 6 and 10 of culture, oocytes were isolated from these follicles and measured their diameters (b). The oocytes increased their sizes in all conditions on Day 6 (C, A vs M, M+A; *P*<0.05). On Day 10, the isolated oocytes similarly reached full-size regardless of the conditions (A vs M, M+A; M vs M+A; *P*<0.05). All bar graphs show the mean ± SD.

**Fig 3:**
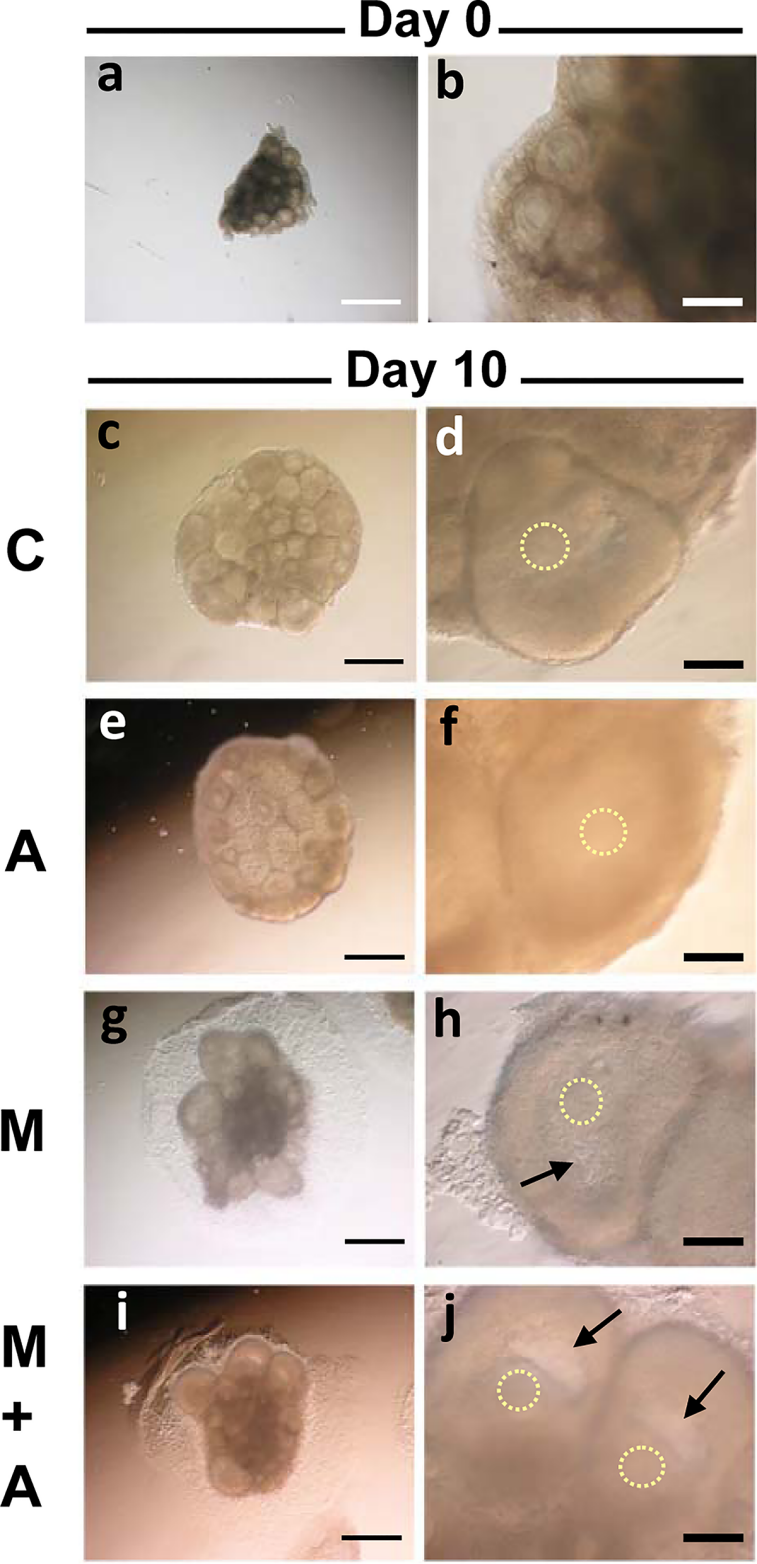
Morphology of ovarian tissues cultured under 4 different conditions. **(a, b)** A whole murine ovary at 14 days old was cut into 8 pieces before culture (a). The most advanced follicles in the tissue was at the preantral stage (b). (**c-j)** Ovarian tissues after 10 days of culture in the C (c, d), A (e, f), M (g, h) and M+A (i, j) conditions. The antral cavity formation occurred only in the follicles under the 3-D Culture system (M, M+A) (arrows). The circles of dots represent the localization of oocytes. Regular bar = 500 μm. Bold bar = 100 μm.

In the 14 dpp murine ovary, most advanced follicles are in the multilayered secondary follicle stage. We first focused on the developmental capacity of secondary follicles under our four different culture conditions. Ovarian tissue pieces were directly placed on a membrane (Membrane culture) and cultured with (A condition) or without (C condition) activin A (Fig 1). Other ovarian tissue pieces were placed on top of a Matrigel drop (3-D culture) and cultured with (M+A condition) or without (M condition) activin A treatment (Fig 1). All tissues were cultured for up to 10 days. We first examined follicle growth by measuring follicle diameters. Before culture (Day 0), the average diameter of the secondary follicles was 131 μm. During tissue culture, the secondary follicles gradually increased in size and expanded granulosa cell layers in all culture conditions. On Day 6 of culture, the average diameters of well-developed follicles at the preantral stage were similar among the four different culture conditions (271~290 μm) (Fig 2A and S1 Table). On Day 10 of culture, the ovarian tissues dramatically increased their size under all four conditions (Fig 3A–3J). Each tissue piece contained 3–10 large follicles and average follicle sizes reached 299–356 μm, although the follicles in the C condition were larger compared to those grown in the other three conditions (*p*<0.05) (Fig 2A and S1 Table).

Next, we examined the growth of germinal vesicle (GV) stage oocytes within the developing follicles cultured under the four different conditions. On Day 6 of culture, oocyte sizes in the 3-D culture systems (M, M+A) were relatively larger than those maintained in the membrane culture systems (C, A) (*p*<0.05) (Fig 2B and S1 Table). On Day 10 of culture, GV oocytes in the larger developed follicles (average diameters of 299–356 um) shown in Fig 2A reached full-size (73.5–76.3 um) (Fig 2B and S1 Table), matching those observed in full-grown GV oocytes in vivo isolated from antral follicles in the 24 dpp ovaries (74.0±1.9 μm in diameter), regardless of the presence or absence of either the Matrigel drop or activin A supplement.

### Histological analysis of ovarian tissues

**Fig 4:**
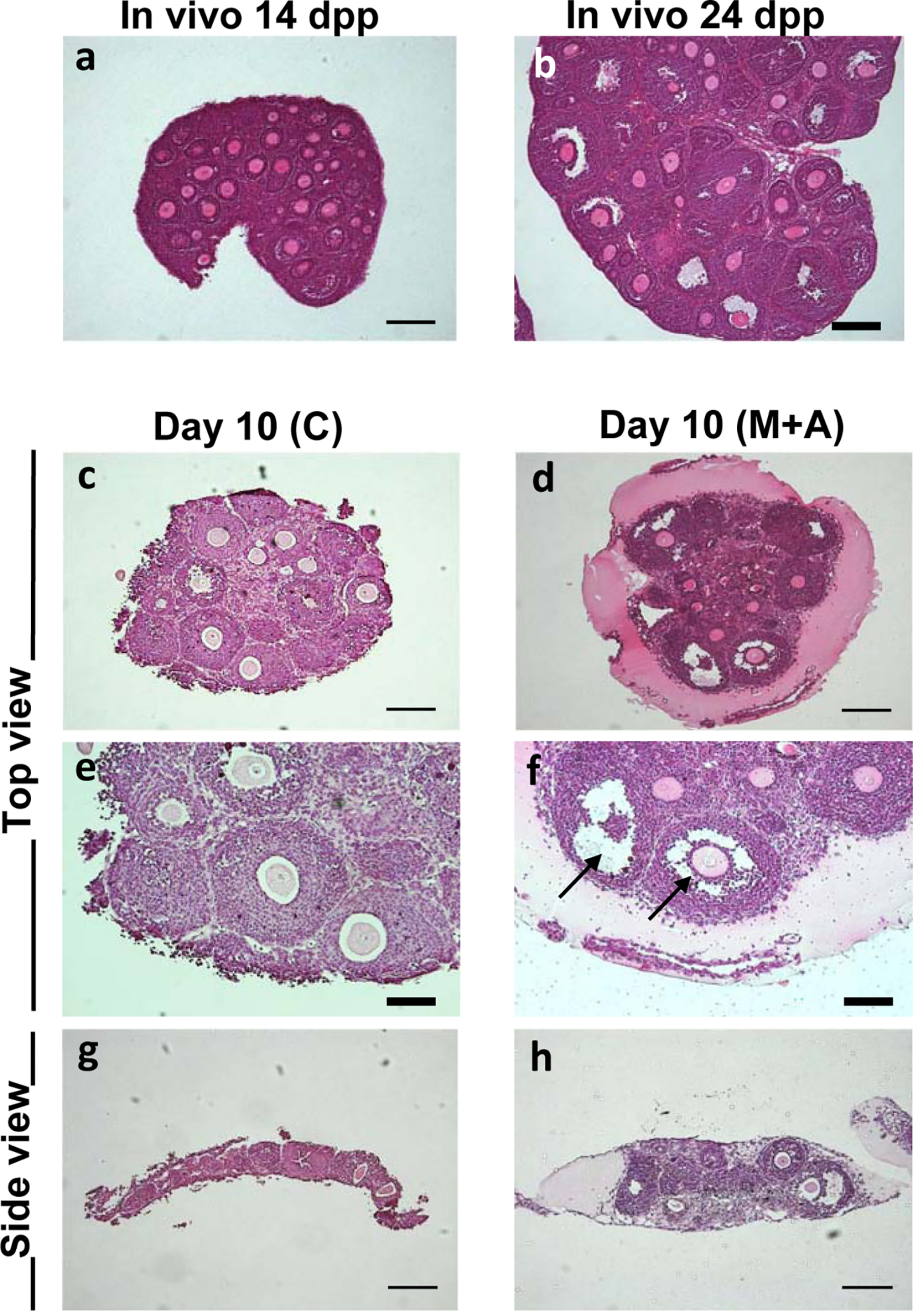
Histological analysis of ovarian tissues under Membrane or 3-D culture system. **(a, b)** Paraffin sections of a whole murine ovary at 14 dpp (a) and 24 dpp (b) with a HE staining. **(c, e, g)** The ovarian tissues cultured in the Membrane culture system (C condition) for 10 days. **(d, f, h)** The ovarian tissues cultured in the 3-D culture system (M+A condition) for 10 days. Follicle growth was detected in both culture systems. Importantly, the antral cavity formation (arrows) occurred only in the 3-D culture system (M, M+A) regardless of the presence or absence of activin A treatment. Regular bar = 200 μm. Bold bar = 100 μm.

To investigate morphological details of follicular development in culture, we prepared paraffin sections of the cultured ovarian tissues (Fig 4). Before culture (Day 0), the 14 dpp in vivo ovary contained multilayered secondary follicles as the most advanced stage follicles (Fig 4A). By 24 dpp, the most advanced follicles had formed antral cavities to develop to antral follicles (Fig 4B). On Day 10 under the membrane culture system (C, A), the largest follicles were observed throughout the tissue without any bias in localization (Fig 4C). These large follicles consisted of multi-layered granulosa cells and full-grown GV oocytes regardless of whether or not activin A was added. Under the 3-D culture system (M, M+A), large follicles were also detected in the tissue, but these were predominantly localized at the periphery of each tissue (Fig 4D), while the central area seemed to undergo gradual degeneration accompanied by loss of healthy follicles after 10 days of culture. Most importantly, however, antral cavity formation occurred only within the large follicles generated in the 3-D culture system (Fig 4D and 4F), and was not observed in follicles cultured in the membrane culture system (Fig 4C and 4E). In the M and M+A conditions, large follicles typically generated an antral cavity to advance their developmental stage regardless of the presence or absence of activin A (Figs 3H, 3J and 4D, 4F). In tissues maintained under the membrane culture conditions, no cavity formation was observed despite steady follicle growth (Figs 3D, 3F and 4C, 4E). In addition, tissues cultured on the Matrigel drop maintained a thickness nearly 3 times larger than those cultured on the membrane filter alone (Fig 4G and 4H), suggesting tissue structure may critically affect follicle development in vitro. These results clearly demonstrate that our 3-D culture system is able to support development of secondary follicles to the antral follicle stage by maintaining normal ovarian architecture.

### Chromatin configuration in GV oocytes grown *in vitro*

**Fig 5:**
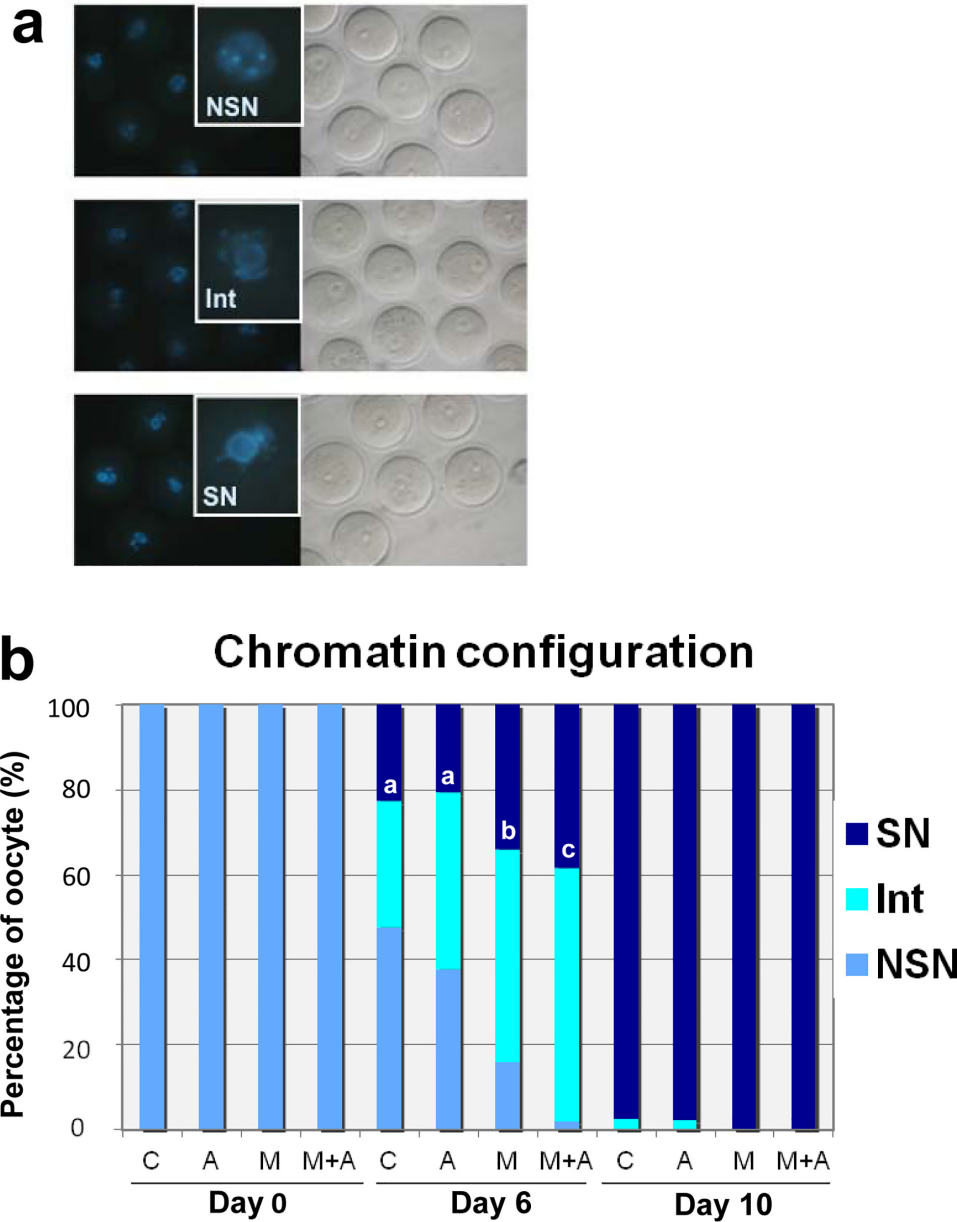
Chromatin configuration of GV oocytes isolated from follicles in ovarian tissues cultured under 4 different conditions. **(a)** Chromatin configurations of mouse GV oocytes after Hoechst staining (400x). By the presence or absence of a ring of condensed chromatin around the nucleorus, the oocytes were categorized into three groups: NSN (non-surrounded nucleolus), SN (surrounded nucleolus) and Int (intermediate stage). The SN configuration represents a stage of GV oocyte that is more advanced toward ovulation. Each square represents a magnified chromatin configuration. **(b)** Distribution of chromatin configuration patterns in the oocytes isolated from the follicles on Days 0, 6 and 10 of culture. On Day 6, the GV chromatin condensation was more advanced under the 3-D Culture (M, M+A) compared to under the Membrane Culture (C, A) (a-c: *P*<0.05; b,c: *P*<0.02). By Day 10 of culture, almost all of full-size GV oocytes progressed to the SN condfiguration regardless of the 4 different culture conditions.

Mammalian oocyte development is characterized by dynamic changes in chromatin structure and function within the germinal vesicle (GV). Chromatin in the GV is initially decondensed with the nucleolus not surrounded by heterochromatin (NSN). During oocyte growth, GV chromatin condenses into perinucleolar rings (SN) [33]. Thus, chromatin condensation provides a good indicator of acquisition of developmental competence in GV oocytes [33]. On the basis of GV chromatin configuration (NSN, Int, SN) (Fig 5A), we assessed nuclear maturity of oocytes grown under the four different culture conditions (Fig 5B). Before culture (Day 0), all GV oocytes isolated from multilayered secondary follicles exhibited the immature NSN chromatin configuration (Fig 5B and S2 Table). On Day 6 after culture, oocytes showed various patterns of chromatin configuration that varied among the culture conditions. In the membrane culture system (C, A condition), 38–48% of oocytes retained the NSN configuration. In contrast, under the 3-D culture without activin A (M), 84% of oocytes had progressed to the Int or SN configuration. In the presence of activin A (M+A), chromatin condensation was further advanced, with almost all oocytes (98%) having reached the Int or SN configuration (Fig 5B and S2 Table). By Day 10 of culture, GV oocytes isolated from the larger follicles had reached full-size as shown in Fig 2B. Almost all of these full-sized GV oocytes had progressed to the nuclear matured status of SN regardless of the culture condition used (Fig 5B and S2 Table).

### IVM

**Fig 6:**
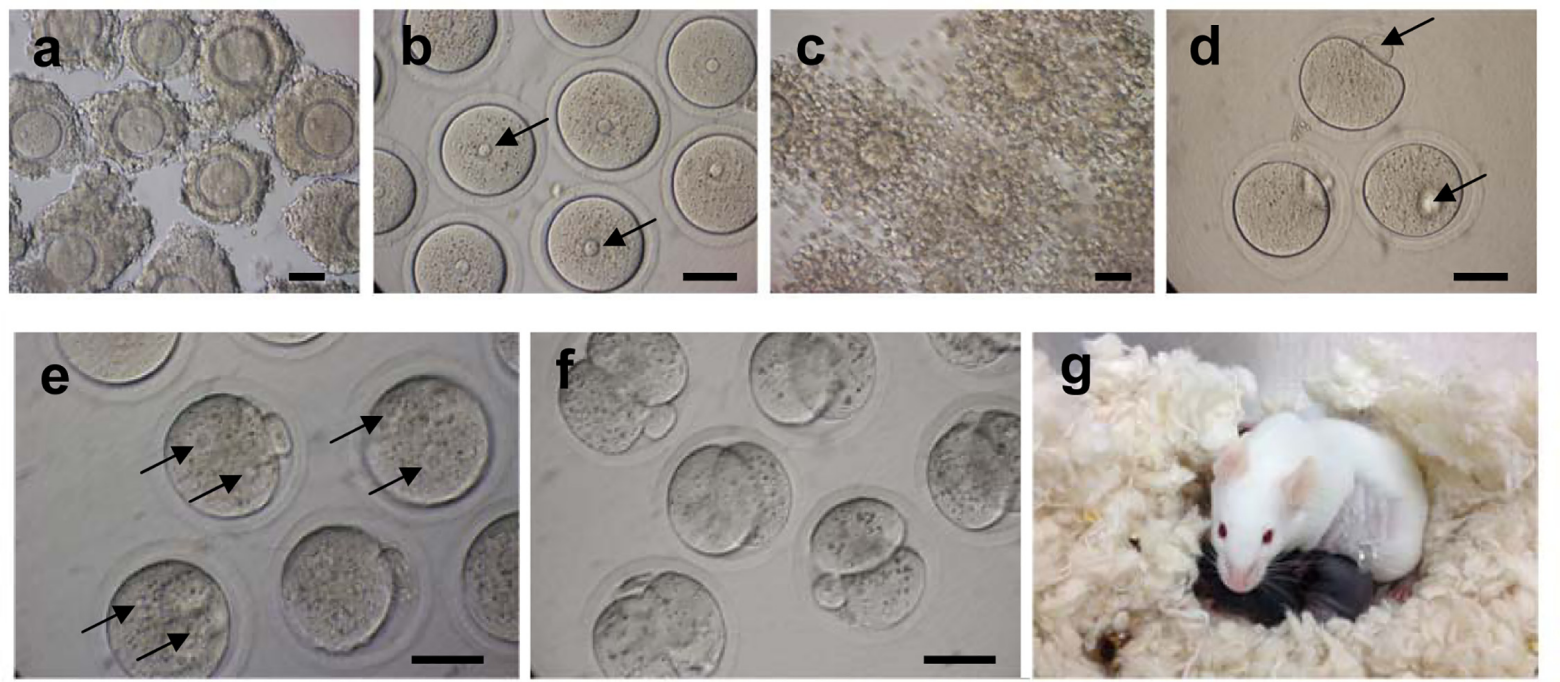
Developmental competence of full-size GV oocytes grown under 3-D culture system. **(a)** After 10 days of the 3-D ovarian tissue culture (M+A), GV oocytes surrounded by granulosa cells were isolated from the antral follicles for in vitro maturation (IVM). **(b)** To observe germinal vesicles (arrows) in GV oocytes, surrounding granulosa cells were mechanically removed. **(c)** After IVM, granulosa cell layers surrounding the oocytes underwent expansion. **(d)** To observe first polar bodies (arrows) in matured MII oocytes, expanded granulosa cell layers were removed after IVM. **(e)** In vitro matured oocytes with expanded granulosa cells were subjected to in vitro fertilization (IVF). Six hrs after IVF, two pronuclei (arrows) were observed in each fertilized oocyte. **(f)** Twenty-four hrs after IVF, some of fertilized oocytes developed to the 2-cell stage embryos. **(g)** The 2-cell embryos were transferred to surrogate mothers to obtain full-term offspring with black coat color. Bar = 40 μm.

We next examined the quality of the full-size GV oocytes grown under the four different conditions. First, we assessed maturation competence of these oocytes (Table 1). On Day 10 of tissue culture, GV oocytes surrounded by granulosa cells were isolated from the larger follicles (Fig 2A and 2B) to be subjected to IVM (Fig 6A and 6B). The GV oocytes in the membrane culture (C, A) were isolated from the follicles without antral cavities (Fig 3D and 3F). However, GV oocytes in the 3-D culture (M, M+A) were collected from the follicles with cavities (Fig 3H and 3J). After IVM, the granulosa cell layers surrounding the oocytes underwent expansion regardless of the culture conditions (Fig 6C). In the membrane culture system (C, A), about 40% of the GV oocytes matured to reach the MII stage in vitro (Fig 6D) regardless of the presence or absence of activin A treatment (Table 1). In the 3-D culture system (M, M+A), in contrast, the maturation rates were significantly increased relative to the membrane culture (Table 1). More than half of the GV oocytes grown under the M condition reached the MII stage in vitro (54%). With the addition of activin A (M+A), the MII rate was further increased to about 66% after IVM.

**Table 1:** *In Vitro* Maturation of GV Oocytes Grown under 4 Different Ovarian Culture Conditions.

		No. of oocytes after *in vitro* maturation			
Condition	No. of GV oocytes (replicates)	Degenerated	GV	GVBD	MII (%)
C	252 (8)	7	13	127	103 (40.9)a
A	60 (3)	0	17	19	24 (40.0)a
M	184 (4)	0	18	65	100 (54.3)b
M+A	262 (6)	2	11	75	172 (65.6)c

Values with different superscripts within the same column are significantly different (a-c: *P*<0.01; b, c: *P*<0.02).

### IVF

After IVM, in vitro matured oocytes surrounded by granulosa cell layers were subjected to IVF and examined for development to the 2-cell embryo stage (Table 2, Fig 6E and 6F). Under the membrane culture without activin A treatment (C), only 11% of the MII oocytes developed to the 2-cell stage. In the presence of activin A (A), the 2-cell rate was slightly increased to 20%. However, using oocytes derived in the 3-D culture system, the 2-cell developmental rates were dramatically improved compared to those derived with membrane culture. Thus, without activin A (M), 45% of 3-D culture system MII oocytes developed to 2-cell embryos after IVF. Activin A treatment (M+A) further improved the developmental capacity of MII oocytes, with 58% developing to the 2-cell stage. These results demonstrate that our 3-D culture system enhances fertilization rates of oocytes grown in vitro, and activin A treatment increases this quality even further.

**Table 2:** Two-Cell Development after *In Vitro* Fertilization of Oocytes Grown under 4 Different Ovarian Culture Conditions.

Condition	No. of MII oocytes (replicates)	No. of 2-cell (%)
C	438 (7)	49 (11.2)a
A	54 (4)	11 (20.4)a
M	118 (3)	53 (44.9)b
M+A	208 (4)	120 (57.7)c

Values with different superscripts within the same column are significantly different (a-c: *P*<0.01; b, c: *P*<0.02).

### ET

To determine the full-term developmental capacity of oocytes derived by IVG, 2-cell stage embryos were transferred into surrogate mothers (Tables 3 and S3). One of forty 2-cell embryos derived from oocytes grown in the membrane culture system without activin A (C) developed into a full-term pup after ET. None of the 2-cell embryos derived from the membrane culture in the presence of activin A (A) developed to full-term pups, suggesting activin A treatment does not, by itself, promote the developmental competence of oocyes grown on a membrane filter. In contrast, oocytes derived from the 3-D culture system supported by a Matrigel drop showed significantly improved rates of full-term development of transferred embryos. Thus, in the absence of activin A (M), we obtained 5 full-term pups from 80 transferred embryos (6%). In the presence of activin A (M+A), developmental competence to term rose to 15%, with 8 full-term pups recovered from 55 transferred embryos (Fig 6G). The average body weights (1.51–1.86 g) and placenta weights (0.15 g) of the pups derived from IVG oocytes were slightly higher than those of in vivo control pups derived from natural mating (Tables 4 and S4). After ET, a number of low-quality embryos derived from IVG oocytes were degenerated to make smaller litters (1–3 pups per surrogate) (S3 and S4 Tables). It has been demonstrated that pups in smaller litters tend to be larger at birth than those in larger litters. All pups were otherwise healthy and normal.

**Table 3:** Newborn Offspring Derived from Oocytes Grown under 4 Different Ovarian Culture Conditions.

Condition	No. of 2-cell embryos transferred (No. of surrogates)	No. of pups (%)
C	40 (5)	1 (2.5)a
A	20 (3)	0 (0)a
M	80 (5)	5 (6.3)a, b
M+A	55 (6)	8 (14.5)b

Values with different superscripts within the same column are significantly different (a, b: *P<*0.0 5).

**Table 4:** Body and Placenta Weights of Newborn Offspring Derived from Oocytes Grown under 4 Different Ovarian Culture Conditions.

Condition	No. of pups	Male/Female	Body weight (g) ± SD	Placenta weight (g) ± SD
C	1	1/0	1.62	0.15
M	5	1/4	1.86 ± 0.38	0.15 ± 0.014
M+A	8	2/6	1.51 ± 0.24	0.15 ± 0.026
*In Vivo* *	7	2/5	1.31 ± 0.08	0.11 ± 0.014

* *In vivo* control pups were derived from natural mating between B6D2F1 female and male mice.

## Discussion

Currently 3-D isolated follicle culture is a popular method to develop secondary ~ preantral follicles to the antral follicle state in vitro. However, this system requires very complicated and time-consuming processes [1, 19, 21–23, 34]. In this study, we have developed a simple and easy ovarian tissue culture system to yield high quality oocytes derived from secondary follicles in mice. A thick Matrigel drop placed under the tissue supports proper follicle development during culture. Oocytes grown in our 3-D culture system acquired much higher developmental competence than those grown without the Matrigel drop, and developed to full-term offspring after IVM and IVF. Activin A supplement further enhanced the developmental competence of oocytes grown in our 3-D ovarian culture system. These results demonstrate that our 3-D culture system provides an optimal environment for proper development of secondary follicles to the antral stage.

In situ ovarian tissue culture is a promising technology for in vitro follicle growth, because this system: i) involves a simple and easy procedure; ii) is not damaged by chemical or mechanical dissociation to isolate follicles; iii) maintains an intact ovarian environment; and iv) could provide an in vitro model for ovarian toxicology testing [35]. Regardless of these advantages, it has been argued that in vitro ovarian culture does not consistently support proper development of advanced follicles [4, 35]. In this study, support afforded by a thick Matrigel drop successfully improved the outcome of ovarian tissue culture to yield developmentally competent oocytes. Thus, it appears the multiple ECM proteins present in Matrigel provide a mechanical scaffold that supports spatial and temporal growth of the multiple follicles within a segment of ovarian tissue. Importantly, the quality of the resulting oocytes was critically improved by our 3-D culture system compared to that detected in oocytes derived from ovarian tissue cultured without the Matrigel drop. These results are consistent with previous reports suggesting that oocytes acquire developmental competence only when the follicle is able to maintain its normal architecture [1]. Normal folliculogenesis is critically regulated by cell-cell communications between granulosa cells and the oocyte, and among granulosa and theca cells through a highly organized network of connections [36]. Our results suggest that a microenvironment conducive to normal folliculogenesis was provided by our 3-D culture system yielding developmentally competent oocytes. Currently encapsulation of follicles in alginate hydrogel is the most widely used 3-D culture system to promote advanced follicle growth [1, 19, 21–23, 34]. Xu et al. (2006) assessed the quality of murine oocytes grown in alginate-encapsulated secondary follicles (150–180 μm) from 16 dpp mice after 8 days in culture [21]. After IVM, about 71% of GV oocytes reached the MII stage, and 68% of those were fertilized after IVF. Four normal offspring (20% of fertilized embryos) were obtained after ET. Our ovarian culture system (M+A condition), yielded success rates of IVM, IVF and ET of 66%, 59%, and 15%, respectively), which were very close to the results reported by Xu et al. (2006), even though our data were derived from younger stage follicles (average: 131 μm, all less than 150 μm in size) recovered from ovaries at 14 dpp and cultured for 10 days, demonstrating that our 3-D culture system reliably generates fertilizable oocytes from secondary follicles in vitro.

In our 3-D system, the tissues have not been enclosed by either Matrigel or the medium, therefore they have easily accessed to air in culture. Recently, alginate encapsulation has been utilized with 3-D ovarian tissue culture to study the growth of the ovarian surface epithelium [37–39]. Small pieces of immature ovary were encapsulated into alginate hydrogel and cultured within medium. As a result, the tissues successfully maintained 3-D structure and grew ovarian epithelium, however, secondary follicles in these tissues did not develop or survive in this culture system [39]. In our preliminary experiment, we also observed that ovarian tissues cultured within medium quickly degenerated and lost all growing follicles (data not shown), suggesting that a hypoxic condition is not optimal for follicle growth in ovarian tissue culture. These observations suggest that optimal culture conditions for follicles within ovarian tissues are quite different than those for isolated follicles. In contrast, it was reported that human cortical ovarian tissues encapsulated within alginate and cultured for six weeks showed at least one primordial follicle that had developed to an antral follicle in the cortex [40]. Taken together, these results suggest that much work remains to be done to completely optimize follicle culture systems specific for each species that can yield normal follicle stages and developmentally competent oocytes in each case.

In this study, we also demonstrated that the activin A treatment further enhances the quality of oocytes grown in 3-D culture. In the ovary, activin A is generated by granulosa cells in primary to antral stage follicles to regulate their proliferation, differentiation, and steroidogenesis [24–25]. As a member of the TGF-β superfamily, activin A stimulates Smad signaling pathways [41] and increases the number of FSH receptors in granulosa cells to promote FSH action on these cells [42]. FSH autocrine signaling allows GTP coupled receptors to activate the PKA pathway leading to granulosa cell differentiation [43]. In our 3-D system, additional activin A treatment appeared to further stimulate these endogenous phenomena to enhance the developmental competence of the resulting oocytes. Zhang *et al*. (2012) found that activin A and preantral granulosa cells cooperated to enhance the growth and maturation of immature oocytes in vitro [44]. Their data further demonstrated that activin A enhances a complex effect on the oocyte maturation, such as oocyte cytoplasmic maturation and oocyte-granulosa cell communication via gap junction [44]. Interestingly, our results showed that addition of activin A to our membrane culture system without Matrigel (our “A condition”) did not enhance oocyte quality, suggesting that exogenous activin A functions only when the ovarian microenvironment is properly organized and maintained. Previous studies have demonstrated that a combination of Matrigel and activin A synergistically enhances in vitro follicle growth compared to conditions utilizing individual ECM proteins plus activin A [8], or Matrigel alone [45]. The ECM proteins act as a reservoir for growth factors, communicate with the intracellular cytoskeleton, and transmit growth factor signals [9, 12]. Indeed, many ECM proteins have binding sites for both growth factors and cell adhesion functions, increasing local concentrations of the growth factors near their cell surface receptors and cell adhesion sites [12]. It was reported that activin A selectively binds to specific ECM proteins such as heparan sulfate proteoglycans [46]. Oktay et al. (2000) demonstrated that an interaction between laminin and activin A promotes the transition from the primary to the multilayer follicle stage, whereas the combination of type IV collagen and activin A suppresses this transition [47]. As previously mentioned, the ovarian microenvironment provides stage-specific ECM composition and regulates actions of multiple factors such as activin A to organize follicle development. Therefore, the interaction between multiple ECM proteins (Matrigel) and activin A might synergistically up-regulate relevant signal transduction pathways in the follicular cells to eventually generate high quality oocytes in vitro. It will be important to determine what ECM component(s) are responsible for enhancing the quality of oocytes in a stage-specific manner.

In summary, the 3-D ovarian culture system we describe here has the potential to be a key technical advance for use in both clinical and laboratory settings. This system can serve as a biological model for studying complex follicle-follicle and follicle-epithelium interactions within the ovary. Furthermore, this model could be useful for toxicological studies involving different animal models both in vivo and in vitro to assess harmful effects of specific compounds or conditions on the female reproductive system including the ovary [48–49]. Currently, reproductive toxicology testing is primarily performed in vivo, however, our culture system could offer a novel in vitro option. For assisted reproductive technologies (ART), the application of in vitro folliculogenesis will contribute to an ultimate goal of the ability to generate fertilizable oocytes in vitro. Especially for prepubertal cancer patients, establishment of this technology will provide a novel approach to fertility preservation. Although it is still very challenging to generate high quality human oocytes in vitro, the 3-D ovarian culture system we describe here for use with mouse tissues will pave the way to advancement of similar technologies enabling fertility preservation in humans.

## Supporting Information

S1 Table Follicle and oocyte growth during the ovarian tissue culture. During the ovarian tissue culture in the membrane (C, A) and in the 3-D (M, M+A) culture systems, we measured the follicle and oocyte sizes on Days 0, 6 and 10 of culture.
(PDF)

S2 Table Chromatin configuration of GV oocytes. On Days 0, 6 and 10 of the ovarian tissue culture, GV oocytes were isolated and subjected to Hoechst staining for 8 minutes at 37°C to determine the status of their chromatin. The GV oocytes were categorized into NSN, Int and SN.
(PDF)

S3 Table Newborn offspring after 2-cell embryo transfer. The 2-cell stage embryos derived from 4 different culture conditions were transferred into the surrogate mothers to obtain full-term pups.
(PDF)

S4 Table Body and placenta weights of newborn offspring. The body and placenta weights of newborn pups were examined and compared to those of *in vivo* derived pups.
(PDF)

## References

[1] Belli M, Vigone G, Merico V, Redi CA, Zuccotti M, Garagna S. Towards a 3D culture of mouse ovarian follicles. Int J Dev Biol 2012; 56: 931–937. 10.1387/ijdb.120175mz 23417415

[2] Matzuk MM, Burns KH, Viveiros MM, Eppig JJ. Intercellular communication in the mammalian ovary: oocytes carry the conversation. Science 2002; 296: 2178–2180. 12077402 10.1126/science.1071965

[3] Luciano AM, Franciosi F, Modina SC, Lodde V. Gap junction-mediated communications regulate chromatin remodeling during bovine oocyte growth and differentiation through cAMP-dependent mechanism(s). Biol Reprod 2011; 85: 1252–1259. 10.1095/biolreprod.111.092858 21816847

[4] Desai N, Alex A, AbdelHafez F, Calabro A, Goldfarb J, Fleischman A, et al Three-dimensional in vitro follicle growth: overview of culture models, biomaterials, design parameters and future directions. Reprod Biol Endocrinol 2010; 8: 119 10.1186/1477-7827-8-119 20946661 PMC2967553

[5] Eppig JJ, Schroeder AC. Capacity of mouse oocytes from preantral follicles to undergo embryogenesis and development to live young after growth, maturation, and fertilization in vitro. Biol Reprod 1989; 41: 268–276. 2508774 10.1095/biolreprod41.2.268

[6] Eppig JJ, O'Brien MJ. Development in vitro of mouse oocytes from primordial follicles. Biol Reprod 1996; 54: 197–207. 8838017 10.1095/biolreprod54.1.197

[7] Hovatta O, Silye R, Abir R, Krausz T, Winston RM. Extracellular matrix improves survival of both stored and fresh human primordial and primary ovarian follicles in long-term culture. Hum Reprod 1997; 12: 1032–1036. 9194661 10.1093/humrep/12.5.1032

[8] Oktem O, Oktay K. The role of extracellular matrix and activin-A in in vitro growth and survival of murine preantral follicles. Reprod Sci 2007; 14: 358–366. 17644808 10.1177/1933719107303397

[9] Berkholtz CB, Shea LD, Woodruff TK. Extracellular matrix functions in follicle maturation. Semin Reprod Med 2006; 24: 262–269. 16944423 10.1055/s-2006-948575 PMC2648384

[10] Figueiredo JR, Hulshof SC, Thiry M, Van den Hurk R, Bevers MM, Nusgens B, et al Extracellular matrix proteins and basement membrane: their identification in bovine ovaries and significance for the attachment of cultured preantral follicles. Theriogenology 1995; 43: 845–858. 16727675 10.1016/0093-691x(95)00036-8

[11] Rodgers RJ, van Wezel IL, Irving-Rodgers HF, Lavranos TC, Irvine CM, Krupa M. Roles of extracellular matrix in follicular development. J Reprod Fertil Suppl 1999; 54: 343–352. 10692866

[12] Kim SH, Turnbull J, Guimond S. Extracellular matrix and cell signalling: the dynamic cooperation of integrin, proteoglycan and growth factor receptor. J Endocrinol 2011; 209: 139–151. 10.1530/JOE-10-0377 21307119

[13] Lee VH, Britt JH, Dunbar BS. Localization of laminin proteins during early follicular development in pig and rabbit ovaries. J Reprod Fertil 1996; 108: 115–122. 8958837 10.1530/jrf.0.1080115

[14] Rodgers HF, Irvine CM, van Wezel IL, Lavranos TC, Luck MR, Sado Y, et al Distribution of the alpha1 to alpha6 chains of type IV collagen in bovine follicles. Biol Reprod 1998; 59: 1334–1341. 9828176 10.1095/biolreprod59.6.1334

[15] van Wezel IL, Rodgers HF, Rodgers RJ. Differential localization of laminin chains in bovine follicles. J Reprod Fertil 1998; 112: 267–278. 9640266 10.1530/jrf.0.1120267

[16] Berkholtz CB, Lai BE, Woodruff TK, Shea LD. Distribution of extracellular matrix proteins type I collagen, type IV collagen, fibronectin, and laminin in mouse folliculogenesis. Histochem Cell Biol 2006; 126: 583–592. 16758163 10.1007/s00418-006-0194-1 PMC2659665

[17] Filatov MA, Khramova YV, Semenova ML. In Vitro Mouse Ovarian Follicle Growth and Maturation in Alginate Hydrogel: Current State of the Art. Acta Naturae 2015; 7: 48–56. PMC4463412 26085944

[18] Pangas SA, Saudye H, Shea LD, Woodruff TK. Novel approach for the three-dimensional culture of granulosa cell-oocyte complexes. Tissue Eng 2003; 9: 1013–1021. 14633385 10.1089/107632703322495655

[19] Xu M, West E, Shea LD, Woodruff TK. Identification of a stage-specific permissive in vitro culture environment for follicle growth and oocyte development. Biol Reprod 2006; 75: 916–923. 16957022 10.1095/biolreprod.106.054833

[20] West ER, Xu M, Woodruff TK, Shea LD. Physical properties of alginate hydrogels and their effects on in vitro follicle development. Biomaterials 2007; 28: 4439–4448. 17643486 10.1016/j.biomaterials.2007.07.001 PMC2034204

[21] Xu M, Kreeger PK, Shea LD, Woodruff TK. Tissue-engineered follicles produce live, fertile offspring. Tissue Eng 2006; 12: 2739–2746. 17518643 10.1089/ten.2006.12.2739 PMC2648391

[22] Jin SY, Lei L, Shikanov A, Shea LD, Woodruff TK. A novel two-step strategy for in vitro culture of early-stage ovarian follicles in the mouse. Fertil Steril 2010; 93: 2633–2639. 10.1016/j.fertnstert.2009.10.027 20004373 PMC2873094

[23] Parrish EM, Siletz A, Xu M, Woodruff TK, Shea LD. Gene expression in mouse ovarian follicle development in vivo versus an ex vivo alginate culture system. Reproduction 2011; 142: 309–318. 10.1530/REP-10-0481 21610168 PMC3145246

[24] Rabinovici J, Spencer SJ, Doldi N, Goldsmith PC, Schwall R, Jaffe RB. Activin-A as an intraovarian modulator: actions, localization, and regulation of the intact dimer in human ovarian cells. J Clin Invest 1992; 89: 1528–1536. 1569191 10.1172/JCI115745 PMC443025

[25] Welt C, Sidis Y, Keutmann H, Schneyer A. Activins, inhibins, and follistatins: from endocrinology to signaling. A paradigm for the new millennium. Exp Biol Med (Maywood) 2002; 227: 724–752. 12324653 10.1177/153537020222700905

[26] Li R, Phillips DM, Mather JP. Activin promotes ovarian follicle development in vitro. Endocrinology 1995; 136: 849–856. 7867593 10.1210/endo.136.3.7867593

[27] Yokota H, Yamada K, Liu X, Kobayashi J, Abe Y, Mizunuma H, et al Paradoxical action of activin A on folliculogenesis in immature and adult mice. Endocrinology 1997; 138: 4572–4576. 9348180 10.1210/endo.138.11.5526

[28] Smitz J, Cortvrindt R, Hu Y, Vanderstichele H. Effects of recombinant activin A on in vitro culture of mouse preantral follicles. Mol Reprod Dev 1998; 50: 294–304. 9621305 10.1002/(SICI)1098-2795(199807)50:3<294::AID-MRD5>3.0.CO;2-E

[29] Zhao J, Taverne MA, van der Weijden GC, Bevers MM, van den Hurk R. Effect of activin A on in vitro development of rat preantral follicles and localization of activin A and activin receptor II. Biol Reprod 2001; 65: 967–977. 11514365 10.1095/biolreprod65.3.967

[30] Telfer EE, McLaughlin M, Ding C, Thong KJ. A two-step serum-free culture system supports development of human oocytes from primordial follicles in the presence of activin. Hum Reprod 2008; 23: 1151–1158. 10.1093/humrep/den070 18326514

[31] Thomas FH, Armstrong DG, Telfer EE. Activin promotes oocyte development in ovine preantral follicles in vitro. Reprod Biol Endocrinol 2003; 1: 76 14613548 10.1186/1477-7827-1-76 PMC280721

[32] Kleinman HK, Martin GR. Matrigel: basement membrane matrix with biological activity. Semin Cancer Biol 2005; 15: 378–386. 15975825 10.1016/j.semcancer.2005.05.004

[33] Tan JH, Wang HL, Sun XS, Liu Y, Sui HS, Zhang J. Chromatin configurations in the germinal vesicle of mammalian oocytes. Mol Hum Reprod 2009; 15: 1–9. 10.1093/molehr/gan069 19019837

[34] West ER, Shea LD, Woodruff TK. Engineering the follicle microenvironment. Semin Reprod Med 2007; 25: 287–299. 17594609 10.1055/s-2007-980222 PMC2648402

[35] Picton HM, Harris SE, Muruvi W, Chambers EL. The in vitro growth and maturation of follicles. Reproduction 2008; 136: 703–715. 10.1530/REP-08-0290 19074213

[36] Kidder GM, Mhawi AA. Gap junctions and ovarian folliculogenesis. Reproduction 2002; 123: 613–620. 12006089 10.1530/rep.0.1230613

[37] Jackson KS, Inoue K, Davis DA, Hilliard TS, Burdette JE. Three-dimensional ovarian organ culture as a tool to study normal ovarian surface epithelial wound repair. Endocrinology 2009; 150: 3921–3926. 10.1210/en.2008-1674 19423762 PMC2717856

[38] King SM, Modi DA, Eddie SL, Burdette JE. Insulin and insulin-like growth factor signaling increases proliferation and hyperplasia of the ovarian surface epithelium and decreases follicular integrity through upregulation of the PI3-kinase pathway. J Ovarian Res 2013; 6: 12 10.1186/1757-2215-6-12 23388061 PMC3724505

[39] King SM, Quartuccio S, Hilliard TS, Inoue K, Burdette JE. Alginate hydrogels for three-dimensional organ culture of ovaries and oviducts. J Vis Exp 2011. 10.3791/2804 PMC3197054 21712801

[40] Laronda MM, Duncan FE, Hornick JE, Xu M, Pahnke JE, Whelan KA, et al Alginate encapsulation supports the growth and differentiation of human primordial follicles within ovarian cortical tissue. J Assist Reprod Genet 2014; 31: 1013–1028. 10.1007/s10815-014-0252-x 24845158 PMC4130945

[41] Heldin CH, Miyazono K, ten Dijke P. TGF-beta signalling from cell membrane to nucleus through SMAD proteins. Nature 1997; 390: 465–471. 9393997 10.1038/37284

[42] Xiao S, Robertson DM, Findlay JK. Effects of activin and follicle-stimulating hormone (FSH)-suppressing protein/follistatin on FSH receptors and differentiation of cultured rat granulosa cells. Endocrinology 1992; 131: 1009–1016. 1505447 10.1210/endo.131.3.1505447

[43] Das S, Maizels ET, DeManno D, St Clair E, Adam SA, Hunzicker-Dunn M. A stimulatory role of cyclic adenosine 3',5'-monophosphate in follicle-stimulating hormone-activated mitogen-activated protein kinase signaling pathway in rat ovarian granulosa cells. Endocrinology 1996; 137: 967–974. 8603610 10.1210/endo.137.3.8603610

[44] Zhang ZP, Liang GJ, Zhang XF, Zhang GL, Chao HH, Li L, et al Growth of mouse oocytes to maturity from premeiotic germ cells in vitro. PLoS One 2012; 7: e41771 10.1371/journal.pone.0041771 22848595 PMC3404094

[45] Guzel Y, Nur Sahin G, Sekeroglu M, Deniz A. Recombinant activin A enhances the growth and survival of isolated preantral follicles cultured three-dimensionally in extracellular basement matrix protein (matrigel) under serum-free conditions. Gynecol Endocrinol 2014; 30: 388–391. 10.3109/09513590.2014.888411 24665930

[46] Li S, Shimono C, Norioka N, Nakano I, Okubo T, Yagi Y, et al Activin A binds to perlecan through its pro-region that has heparin/heparan sulfate binding activity. J Biol Chem 2010; 285: 36645–36655. 10.1074/jbc.M110.177865 20843788 PMC2978593

[47] Oktay K, Karlikaya G, Akman O, Ojakian GK, Oktay M. Interaction of extracellular matrix and activin-A in the initiation of follicle growth in the mouse ovary. Biol Reprod 2000; 63: 457–461. 10906050 10.1095/biolreprod63.2.457

[48] Tiemann U. In vivo and in vitro effects of the organochlorine pesticides DDT, TCPM, methoxychlor, and lindane on the female reproductive tract of mammals: a review. Reprod Toxicol 2008; 25: 316–326. 10.1016/j.reprotox.2008.03.002 18434086

[49] Stefansdottir A, Fowler PA, Powles-Glover N, Anderson RA, Spears N. Use of ovary culture techniques in reproductive toxicology. Reprod Toxicol 2014; 49C: 117–135. 10.1016/j.reprotox.2014.08.001 25150138

