# Supplementary material for: A Simplified Method for Three-Dimensional (3-D) Ovarian Tissue Culture Yielding Oocytes Competent to Produce Full-Term Offspring in Mice
Source: PLoS One. 2015 Nov 16;10(11):e0143114. doi: 10.1371/journal.pone.0143114 (PMC4646357; doi:10.1371/journal.pone.0143114)
Supplement: S1 Table — During the ovarian tissue culture in the membrane (C, A) and in the 3-D (M, M+A) culture systems, we measured the follicle and oocyte sizes on Days 0, 6 and 10 of culture. (PDF) [file pone.0143114.s001.pdf]

S1 Table. Follicle and oocyte growth during the ovarian tissue culture.

	Day 0			Day 6			Day 10		
Condition	No. of follicles/ oocytes analyzed	Follicle diameter (µm ± SD)	Oocyte diameter (µm ± SD)	No. of follicles/ oocytes analyzed	Follicle diameter (µm ± SD)	Oocyte diameter (µm ± SD)	No. of Follicles/ oocytes analyzed	Follicle diameter (µm ± SD)	Oocyte diameter (µm ± SD)
C	145	130.9 ± 18.5	58.6 ± 4.5	165	270.8 ± 48.5	67.2 ± 4.0	165	356.0 ± 44.8	73.5 ± 4.1
A				15	286.7 ± 25.4	68.6 ± 1.1	15	299.2 ± 22.6	73.9 ± 2.3
M				60	289 ± 35.2	71.3 ± 3.4	60	309.6 ± 30.8	74.1 ± 3.9
M+A				75	290.4 ± 36.8	72.8 ± 2.9	75	335.1 ± 31.3	76.3 ± 2.8
