# Supplementary material for: A Simplified Method for Three-Dimensional (3-D) Ovarian Tissue Culture Yielding Oocytes Competent to Produce Full-Term Offspring in Mice
Source: PLoS One. 2015 Nov 16;10(11):e0143114. doi: 10.1371/journal.pone.0143114 (PMC4646357; doi:10.1371/journal.pone.0143114)
Supplement: S2 Table — On Days 0, 6 and 10 of the ovarian tissue culture, GV oocytes were isolated and subjected to Hoechst staining for 8 minutes at 37°C to determine the status of their chromatin. The GV oocytes were categorized into NSN, Int and SN. (PDF) [file pone.0143114.s002.pdf]

S2 Table. Chromatin configuration of GV oocytes.

Day of analysis	Condition	Total No. of oocytes	Chromatin Configuration		
			NSN (%)	Int (%)	SN (%)
Day 0	C	45	45 (100)	0	0
	A				
	M				
	M+A				
Day 6	C	44	21 (47.7)	13 (29.5)	10 (22.7)
	A	29	11 (37.9)	12 (41.4)	6 (20.7)
	M	44	7 (15.9)	22 (50.0)	15 (34.1)
	M+A	47	1 (2.1)	28 (59.6)	18 (38.3)
Day 10	C	39	0	1 (2.6)	38 (97.4)
	A	41	0	1 (2.4)	40 (97.6)
	M	45	0	0	45 (100)
	M+A	44	0	0	44 (100)

NSN: Non Surrounded Nucleolus
Int: Intermediate stage
SN: Surrounded Nucleolus
