# Supplementary material for: A Simplified Method for Three-Dimensional (3-D) Ovarian Tissue Culture Yielding Oocytes Competent to Produce Full-Term Offspring in Mice
Source: PLoS One. 2015 Nov 16;10(11):e0143114. doi: 10.1371/journal.pone.0143114 (PMC4646357; doi:10.1371/journal.pone.0143114)
Supplement: S3 Table — The 2-cell stage embryos derived from 4 different culture conditions were transferred into the surrogate mothers to obtain full-term pups. (PDF) [file pone.0143114.s003.pdf]

S3 Table. Newborn offspring after 2-cell embryo transfer.

C (Control)

No. of Exp.	No. of 2-cell embryos transferred (No. of surrogates)	No. of pups
1	15 (2)	0
2	15 (2)	0
3	10 (1)	1
Total	40 (5)	1 (2.5%)

A (Activin)

No. of Exp.	No. of 2-cell embryos transferred (No. of surrogates)	No. of pups
1	7 (1)	0
2	6 (1)	0
3	7 (1)	0
Total	20 (3)	0

M (Matrigel)

No. of Exp.	No. of 2-cell embryos transferred (No. of surrogates)	No. of pups
1	15 (1)	0
2	38 (2)	3
3	9 (1)	1
4	18 (1)	1
Total	80 (5)	5 (6.3%)

M+A (Matrigel + Activin A)

No. of Exp.	No. of 2-cell embryos transferred (No. of surrogates)	No. of pups
1	17 (2)	4
2	17 (2)	2
3	10 (1)	1
4	11 (1)	1
Total	55 (6)	8 (14.5%)
