# Supplementary material for: A Simplified Method for Three-Dimensional (3-D) Ovarian Tissue Culture Yielding Oocytes Competent to Produce Full-Term Offspring in Mice
Source: PLoS One. 2015 Nov 16;10(11):e0143114. doi: 10.1371/journal.pone.0143114 (PMC4646357; doi:10.1371/journal.pone.0143114)
Supplement: S4 Table — The body and placenta weights of newborn pups were examined and compared to those of in vivo derived pups. (PDF) [file pone.0143114.s004.pdf]

S4 Table. Body and placenta weights of newborn offspring.

C (Control)

No. of Exp. *	Sex	Body weight (g)	Placenta weight (g)
3	M	1.62	0.15

* No. of Exp. of ET (see Table S4).

A (Activin)

No full-term pup was obtained.

M (Matrigel)

No. of Exp. *	Sex	Body weight (g)	Placenta weight (g)
2	F	1.35	0.13
2	F	2.4	0.16
2	F	2.14	0.16
3	F	1.54	0.16
4	M	1.89	0.14
Average ± SD		1.86 ± 0.38	0.15 ± 0.014

* No. of Exp. of ET (see Table S4).

M+A (Matrigel + Activin A)

No. of Exp. *	Sex	Body weight (g)	Placenta weight (g)
1	F	1.62	0.15
1	F	1.56	0.14
1	F	1.64	0.15
1	M	1.43	0.16
2	F	1.76	0.18
2	F	1.36	0.14
3	M	1.02	0.1
4	F	1.71	0.18
Average ± SD		1.51 ± 0.24	0.15 ± 0.026

***** No. of Exp. of ET (see Table S4).

.

In Vivo Control (natural mating)

No. of Exp.**	Sex	Body weight (g)	Placenta weight (g)
1	F	1.3	0.14
1	F	1.26	0.14
1	M	1.46	0.12
1	F	1.32	0.1
1	M	1.35	0.11
1	F	1.24	0.1
1	F	1.24	0.09
Average ± SD		1.31 ± 0.08	0.11 ± 0.014

****** Derived from one litter.
